# Selective Hypoxia-Sensitive Oxomer Formation by FIH Prevents Binding of the NF-κB Inhibitor IκBβ to NF-κB Subunits

**DOI:** 10.1080/10985549.2024.2338727

**Published:** 2024-04-22

**Authors:** Yulia L. Volkova, Agnieszka E. Jucht, Nina Oechsler, Roopesh Krishnankutty, Alex von Kriegsheim, Roland H. Wenger, Carsten C. Scholz

**Affiliations:** a Institute of Physiology, University of Zurich, Zurich, Switzerland; b Institute of Physiology, University Medicine Greifswald, Greifswald, Germany; c Institute of Genetics and Cancer, University of Edinburgh, UK

**Keywords:** HIF1AN, HIF, hydroxylase inhibitor, inflammation, oxygen sensor

## Abstract

Pharmacologic inhibitors of cellular hydroxylase oxygen sensors are protective in multiple preclinical *in vivo* models of inflammation. However, the molecular mechanisms underlying this regulation are only partly understood, preventing clinical translation. We previously proposed a new mechanism for cellular oxygen sensing: oxygen-dependent, (likely) covalent protein oligomer (oxomer) formation. Here, we report that the oxygen sensor factor inhibiting HIF (FIH) forms an oxomer with the NF-κB inhibitor β (IκBβ). The formation of this protein complex required FIH enzymatic activity and was prevented by pharmacologic inhibitors. Oxomer formation was highly hypoxia-sensitive and very stable. No other member of the IκB protein family formed an oxomer with FIH, demonstrating that FIH-IκBβ oxomer formation was highly selective. In contrast to the known FIH-dependent oxomer formation with the deubiquitinase OTUB1, FIH-IκBβ oxomer formation did not occur via an IκBβ asparagine residue, but depended on the amino acid sequence VAERR contained within a loop between IκBβ ankyrin repeat domains 2 and 3. Oxomer formation prevented IκBβ from binding to its primary interaction partners p65 and c-Rel, subunits of NF-κB, the master regulator of the cellular transcriptional response to pro-inflammatory stimuli. We therefore propose that FIH-mediated oxomer formation with IκBβ contributes to the hypoxia-dependent regulation of inflammation.

## Introduction

Decreased oxygen availability (hypoxia) and inflammation frequently co-occur with mutual functional effects.^1^ Cells adapt to hypoxia via cellular oxygen sensors, including prolyl-4-hydroxylase domain (PHD) proteins 1–3 and factor inhibiting HIF (FIH).^2^ FIH was first described to catalyze the hydroxylation of an asparagine residue within α subunits of the heterodimeric hypoxia-inducible factor (HIF),^3–6^ inhibiting the interaction of HIF with the transcriptional co-activators p300 and CBP histone acetyl transferases.^7^

Pharmacologic hydroxylase inhibitors (HIs) are protective in animal models of inflammation, including inflammatory bowel disease, sepsis, lung inflammation, skin abscess, chronic kidney disease, bladder infection and more.^8–10^ However, the underlying molecular mechanisms of the HI’s protective effects are scarcely understood.^11–13^ FIH has recently been linked to the regulation of inflammation *in vivo*. Treatment with the FIH-selective pharmacologic agent N-oxalyl-D-phenylalanine (NOFD) was protective against irradiation-induced injuries of the gastrointestinal tract and FIH deletion in the colon epithelium attenuated chronic colitis.^14^^,^^15^ We previously demonstrated that the enzymatic activities of FIH and PHD1 attenuate the major pro-inflammatory IL-1β signaling pathway.^12^ Nonetheless, the general relevance and the detailed FIH-dependent regulation of inflammation remains elusive.

FIH enzymatic activity is not limited to asparagine hydroxylation or HIF-α subunits.^16^ The first FIH substrates identified outside the HIF pathway were IκBα and p105, both members of the inhibitor of NF-κB (IκBs) protein family.^17^ The IκB protein family can be separated into three different groups: typical (IκBα, IκBβ and IκBε), precursor (p105/IκBγ and p100/IκBδ) and atypical (nuclear) IκBs (Bcl-3, IκBζ, IκBNS, and IκBη).^18^^,^^19^ In the absence of a stimulus, NF-κB is retained in the cytoplasm by interaction with typical IκB proteins.^18^ Following an inflammatory stimulus, typical IκBs are phosphorylated and degraded, releasing NF-κB which then translocates into the nucleus and upregulates the transcription of specific genes.^20^^,^^21^ In addition to the hydroxylation of IκBα and p105, IκBε has previously been shown to interact with FIH.^22^ Of note, all members of the IκB protein family contain ankyrin repeat domains (ARDs) and proteins with ARDs represent the largest group among the known FIH substrates.^17^^,^^22^^,^^23^ Thus, there is strong link between FIH and IκB proteins, although the hydroxylation of IκB proteins is apparently not affecting IκBs’ function.^17^^,^^24^

We recently reported that the deubiquitinase (DUB) ovarian tumor domain-containing ubiquitin aldehyde binding 1 (OTUB1) is a substrate for FIH-mediated hydroxylation of asparagine residue N22.^12^^,^^25^ In addition, FIH and OTUB1 form an oxygen-dependent denaturation-resistant (likely covalent) protein-protein complex catalyzed by FIH activity, which we refer to as “oxygen-dependent stable protein oligomer” (oxomer).^16^^,^^26^^,^^27^ FIH-OTUB1 oxomer formation is highly hypoxia sensitive and regulates OTUB1 enzymatic function.^26^ A mass spectrometry-based analysis identified 12 additional putative stable protein complexes formed by FIH, including ubiquitin-like modifier-activating enzyme 1 (UBA1) and IκBβ.^26^ Interestingly, we had previously identified IκBβ as interaction partner of FIH together with oxidation of aspartate residue 195, indicating a potential hydroxylation of IκBβ by FIH.^12^

Here, we investigated if FIH forms oxomer complexes with other proteins. Due to the existing functional links between FIH and inflammation, we focused on IκB proteins as putative substrates for oxomer formation. The identification of additional oxomer complexes and their characterization will increase our understanding of this novel type of oxygen-dependent signaling. Moreover, characterization of the functional interaction of FIH with proteins of pro-inflammatory signaling pathways will contribute to our understanding of the molecular mechanism(s) underlying the protective effect of pharmacologic FIH inhibition and genetic deletion in inflammatory diseases *in vivo*.

## Results

### FIH forms an oxomer with IκBβ but not with other IκB protein family members

**Figure 1.**
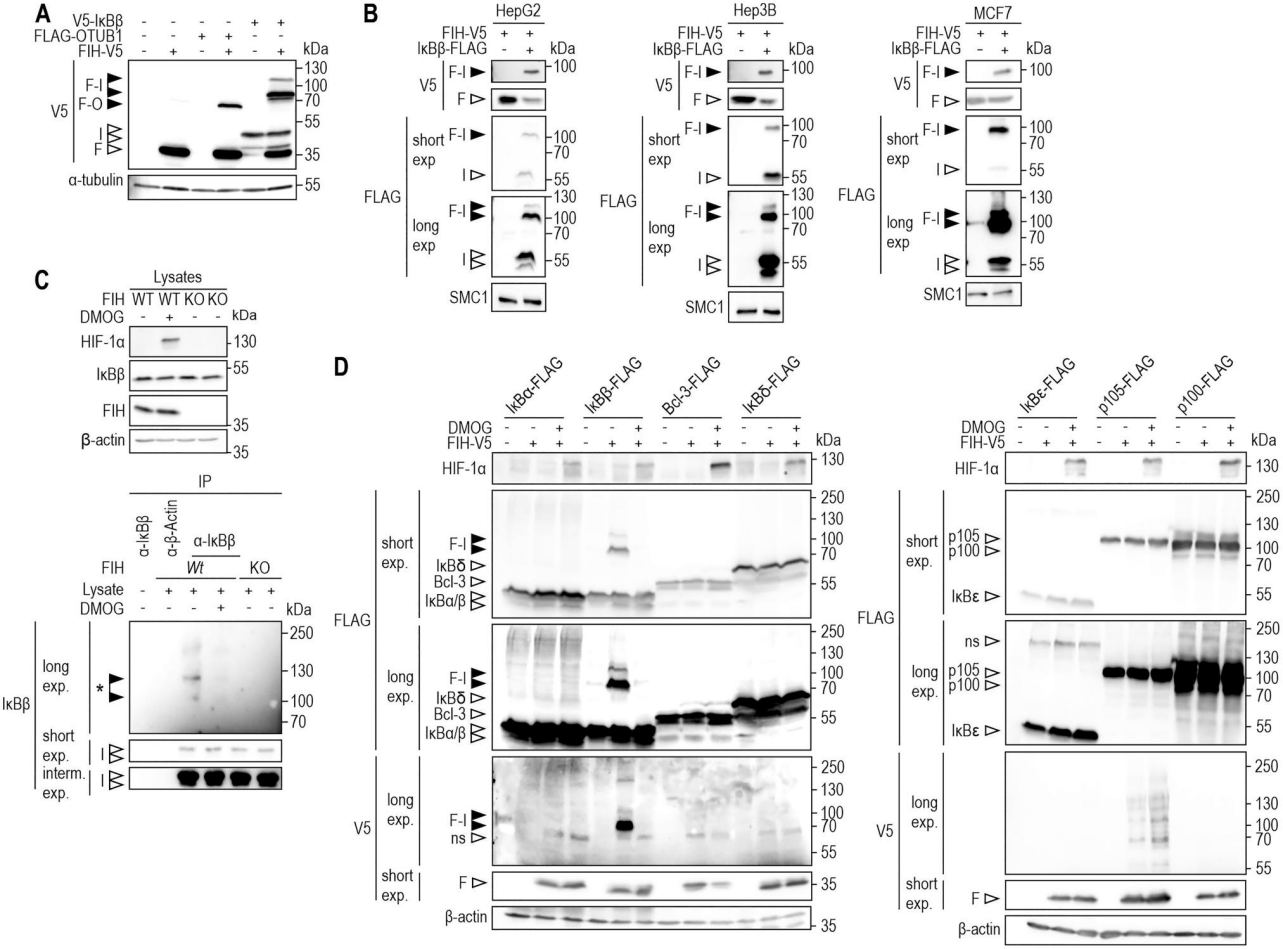
Selective oxomer formation between FIH and IκBβ. (A) Immunoblot analysis of the oxomer formation between ectopically expressed FIH-V5 and V5-IκBβ in HEK293 cells, using FIH-OTUB1 oxomer formation via ectopic expression of FIH-V5 and FLAG-OTUB1 as control. (B) Immunoblot analysis of FIH-IκBβ oxomer formation in HepG2, Hep3B and MCF7 cells with ectopic FIH-V5 and IκBβ-FLAG expression. (C) Immunoprecipitation of endogenous IκBβ from HEK293 cells. A signal was detected at a molecular weight consistent with a FIH-IκBβ oxomer (highlighted by *). This complex was absent in the presence of the hydroxylase inhibitor DMOG (1 mM for 24 h) and in FIH knockout (KO) cells. (D) Immunoblot analysis of oxomer formation between FIH-V5 and the Indicated members of the IκB protein family following transient transfection of HEK293 cells with corresponding vectors for ectopic expression. Predicted molecular weights of putative FIH oxomers (without post-translational protein modifications): FIH-IκBα, 76 kDa; FIH-IκBβ, 78 kDa; FIH-Bcl-3, 88 kDa; FIH-IκBδ, 90 kDa; FIH-IκBε, 78 kDa; FIH-p105, 145 kDa; FIH-p100, 137 kDa. Data are representative of three (A, B, D) and two (C) independent experiments. F, FIH-V5; I, V5-IκBβ; F-O, FIH-OTUB1 oxomer; F-I, FIH-IκBβ oxomer; interm., intermediate; exp, exposure; DMOG, dimethyloxalylglycine; ns, non-specific.

Following the previous identification of 12 potential substrate proteins for oxomer formation with FIH,^26^ we aimed to analyze these candidates in more detail and focused on IκBβ and UBA1. IκBβ was of special interest, as members of its protein family had been shown to be hydroxylated and/or to interact with FIH.^17^^,^^22^^,^^23^ Moreover, we had previously identified IκBβ as interactor of FIH and we found a putative hydroxylation site within IκBβ.^12^ UBA1 was selected for further investigation because it plays a pivotal role in protein ubiquitination as E1 enzyme, initiating the enzymatic ubiquitination cascade.^28^^,^^29^ We thus assessed the oxomer formation between ectopically expressed FIH and IκBβ (Figure 1A) and between FIH and UBA1 (Figure S2) by immunoblotting. Oxomer formation between FIH and OTUB1 served as positive control (Figure 1A).^26^ Ectopic co-expression of UBA1 or IκBβ with FIH led to the formation of denaturing condition-resistant (SDS-PAGE) complexes at molecular weights that were consistent with the covalent addition of UBA1 or IκBβ, respectively, to FIH (Figure 1A and Figure S2). Moreover, a stable FIH-IκBβ complex was also formed in HepG2, Hep3B and MCF7 cells (Figure 1B), suggesting a general occurrence of the observed oxomer formation. Interestingly, the ratio between the FIH-IκBβ oxomer and IκBβ monomer differed between the cell lines with the highest relative FIH-IκBβ oxomer levels being observed in MCF-7 cells (Figure 1B).

Next, we assessed if FIH-IκBβ oxomer formation was also observable between endogenous proteins and whether it was dependent on FIH enzymatic function. Following IP of endogenous IκBβ, a signal was detected at a molecular weight that corresponded to the predicted FIH-IκBβ oxomer (Figure 1C and Figure S3A). This signal was abolished following FIH inhibition by DMOG treatment or FIH gene deletion (Figure 1C and Figure S3A). Thus, also endogenous FIH and IκBβ form a denaturing condition-resistant oxomer which depends on FIH enzymatic activity.

The successful validation of FIH-IκBβ oxomer formation raised the question whether other IκB family members also served as FIH substrates for oxomer formation. To investigate this possibility, FIH was ectopically co-expressed with either IκBα, IκBβ, Bcl-3, IκBδ, IκBε, p105 or p100 and it was assessed if any higher molecular weight signals could be detected that were sensitive to pharmacologic FIH inhibition (Figure 1D) or FIH deletion (Figure S3B). Although several IκB family members had previously been shown to serve as hydroxylation substrates and/or interaction partners of FIH,^17^^,^^22^^,^^23^ none of the other IκB proteins formed an oxomer with FIH (Figure 1D and Figure S3B), demonstrating that FIH-mediated oxomer formation is highly selective for IκBβ.

### FIH-IκBβ oxomer formation is highly sensitive to hypoxia

**Figure 2.**
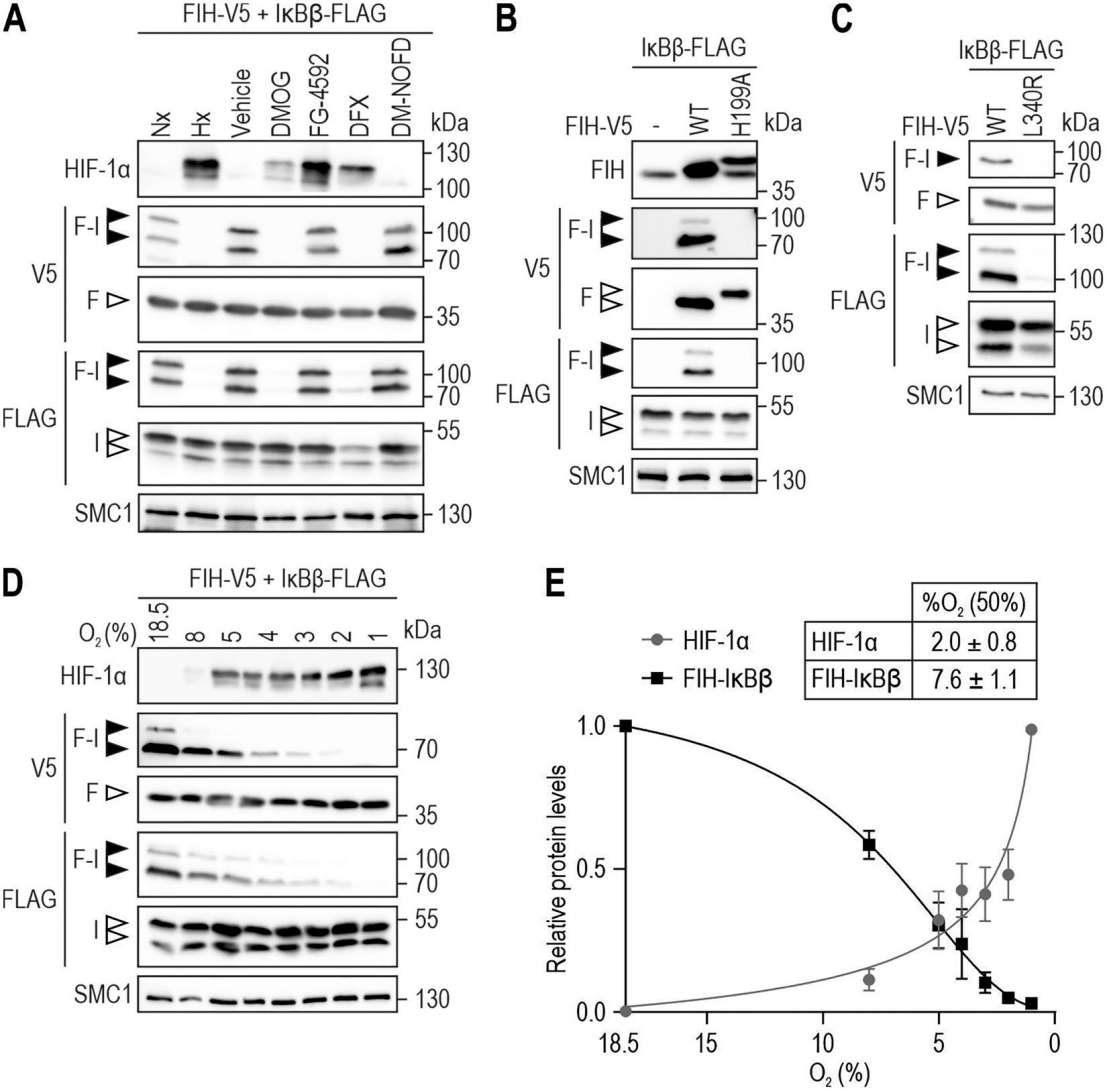
FIH-IκBβ oxomer formation depends on FIH enzymatic activity and is highly hypoxia-sensitive. (A) FIH-IκBβ oxomer formation is prevented by hypoxia (0.2% O_2_), DMOG (1 mM) and DFX (0.1 mM), but not by the PHD-selective FG-4592 (0.1 mM) or the FIH-selective DM-NOFD (1 mM). Treatments were performed for 16 h in HEK293 cells. (B, C) Genetically inactivated FIH (H199A or L340R loss of function mutations) prevented FIH-IκBβ oxomer formation. (D) Hypoxia sensitivity of HIF-1α stabilization and FIH-IκBβ oxomer formation after 24 h at the indicated oxygen levels. (E) Quantification of the results shown in (D) and calculation of the corresponding oxygen sensitivities. Data are shown as mean ± SEM. F, FIH-V5; I, IκBβ-FLAG; Nx, normoxia; Hx, hypoxia (0.2% O_2_); WT, wild-type. Data are representative of three (A-C) and four (D, E) independent experiments.

To investigate the relevance of FIH enzymatic activity for FIH-IκBβ oxomer formation in detail, HEK293 cells were incubated in hypoxia (0.2% O_2_; pan-hydroxylase inhibition) or treated with pan- (DMOG, DFX), PHD- (FG-4592) or FIH-selective (DM-NOFD) hydroxylase inhibitors.^27^^,^^30^ Formation of the FIH-IκBβ oxomer was abrogated by hypoxia, DMOG and DFX but not by FG-4592 (roxadustat) (Figure 2A) in agreement with the reported selectivity of this inhibitor for PHDs.^27^^,^^31^ Interestingly, DM-NOFD did not prevent FIH-IκBβ oxomer formation (Figure 2A), like we previously observed for FIH-OUTB1 oxomer formation.^27^

To investigate the effect of genetic inhibition of FIH activity, cells were used with ectopic expression of FIH carrying a point mutation of the active site aa residue histidine 199 to alanine (H199A).^4^ This point mutation prevented FIH-IκBβ oxomer formation (Figure 2B). Moreover, also the mutation of FIH lysine 340 to arginine, disrupting FIH dimerization and thereby FIH hydroxylase activity,^32^ prevented FIH-IκBβ oxomer formation (Figure 2C). Overall, these results demonstrated that FIH enzymatic activity is required for FIH-IκBβ oxomer formation.

Because FIH-IκBβ oxomer formation required FIH activity, the sensitivity of FIH-IκBβ oxomer formation to different oxygen availability (18.5–1% O_2_) was investigated. Interestingly, FIH-IκBβ oxomer formation was highly sensitive to hypoxia, with an EC_50_ of 7.6% O_2_ compared to an EC_50_ for HIF-1α stabilization of 2.0% O_2_ within the same cells (Figure 2D and E).

### The FIH-IκBβ oxomer is an extraordinarily stable protein complex

**Figure 3.**
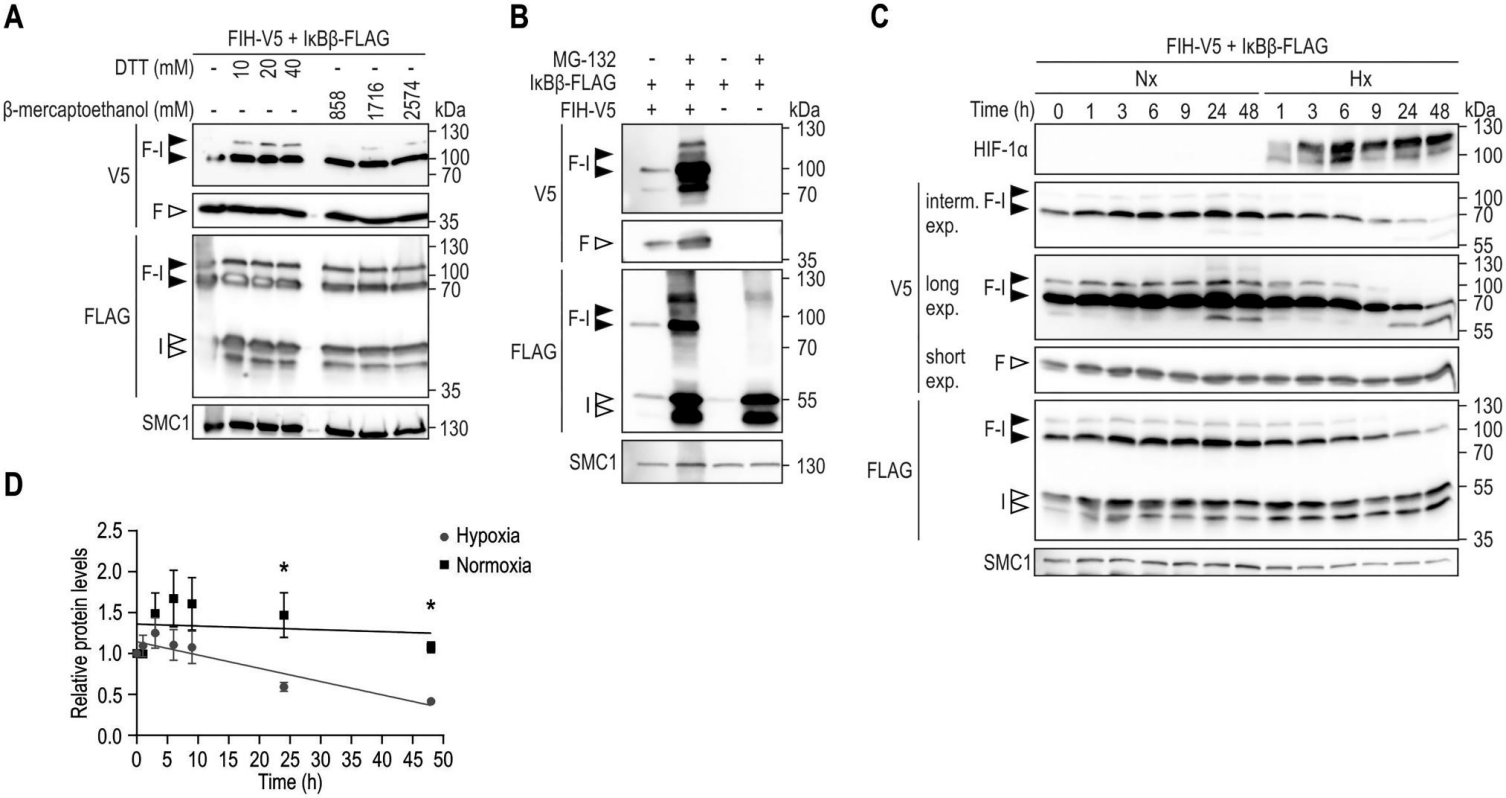
FIH-IκBβ oxomer stability. (A) Immunoblot analysis of the FIH-IκBβ oxomer upon treatment of cell lysates with the indicated reducing agents followed by incubation for 5 min at 95 °C. (B) FIH-IκBβ oxomer formation in HEK293 cells following the indicated transfection and treatment with the proteasome inhibitor MG132 (10 μM). (C) HEK293 cells were transfected with vectors encoding FIH and IκBβ for 22 h to form the FIH-IκBβ oxomer. Subsequently, oxomer stability was analyzed in hypoxia (0.2% O_2_), inhibiting FIH and thus preventing any additional oxomer formation. (D) Quantification of the results shown in (C), presented as mean ± SEM. F, FIH-V5; I, IκBβ-FLAG; F-I, FIH-IκBβ oxomer; DTT, dithiothreitol; interm., intermediate; exp., exposure. *P < 0.05 by Student’s *t* test. Data are representative of three independent experiments.

Given the resistance of the FIH-IκBβ oxomer against the denaturing conditions during immunoblotting, we next characterized the stability of this oxomer in more detail. Recently, a new type of covalent bond between a lysine and a cysteine residue has been described, called a NOS bridge.^33^ The NOS bridge can be disrupted under very strong reducing conditions (1–5 mM dithiothreitol, DTT). To investigate if FIH and IκBβ are linked through a NOS bridge within the observed oxomer, samples were treated with different reducing agents at even higher concentrations than those used for disrupting the NOS bridge (Figure 3A). None of the used concentrations of DTT or β-mercaptoethanol disrupted the FIH-IκBβ oxomer, indicating that the linkage between FIH and IκBβ is not a NOS bridge or any other type of bond that is susceptible to the reducing conditions described.

Next, we analyzed the degradation and stability of the FIH-IκBβ oxomer. To assess if the oxomer was degraded by the proteasome, cells were allowed to form the oxomer for 24 h and subsequently treated with the proteasome inhibitor MG132 for 21 h (Figure 3B). Proteasome inhibition strongly increased FIH, IκBβ and the oxomer levels (Figure 3B), also demonstrating that the oxomer is degraded via the proteasome. In a subsequent experiment, the stability of the oxomer was analyzed. Following oxomer formation for 22 h, cells were exposed to normoxia or hypoxia (0.2% O_2_) for the indicated time periods to prevent any additional oxomers to be formed (Figure 3C). In normoxia, no change of the oxomer protein level was observed over the entire 48 h (Figure 3C and D). In hypoxia, a first significant reduction of the oxomer was detected only 24 h after the start of the hypoxia exposure and reached approximately 50% of the initial levels at around 48 h following the onset of hypoxia (Figure 3C and D).

### The IκBβ loop between ankyrin repeat domains 2 and 3 is necessary for oxomer formation

**Figure 4.**
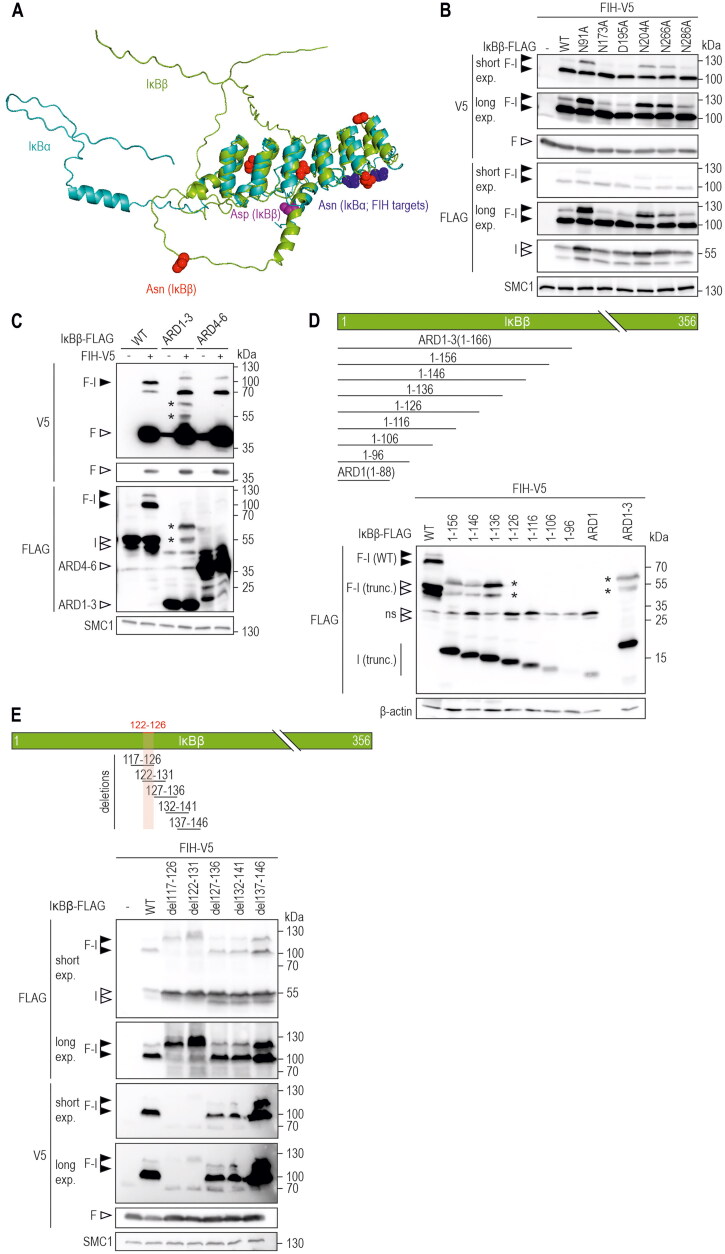
Analysis of potential IκBβ amino acid residues as substrates for FIH-IκBβ oxomer formation. (A) Overlay of protein structures of IκBα (shown in cyan, identifier AF-P25963-F1) and IκBβ (shown in green, identifier AF-Q15653-F1) predicted by AlphaFold Protein Structure Database. Asparagine residues of IκBα previously demonstrated to be hydroxylated by FIH^17^ are indicated in dark blue, all asparagine residues of IκBβ are highlighted in red. An aspartic acid residue that had previously been found to be oxidized is highlighted in magenta.^12^ (B) Separate point mutations were introduced in IκBβ-FLAG, changing each asparagine residue and the highlighted aspartic acid residue to alanine. Oxomer formation was assessed in HEK293 cells by immunoblotting. (C) Analysis of oxomer formation between FIH and truncated IκBβ (ARD 1–3, 1–166 amino acids; ARD 4–6, 167–356 amino acids of full length IκBβ). (D) Analysis of oxomer formation between FIH and the indicated C-terminally truncated IκBβ fragments. (E) Analysis of oxomer formation between FIH and IκBβ containing the indicated amino acid deletions. Amino acids identified to be involved in oxomer formation (122–126) are highlighted in red. F, FIH-V5; I, IκBβ-FLAG; WT, wild-type; ARD, ankyrin repeat domain; *FIH-IκBβ oxomer with truncated IκBβ forms; trunc., truncated; exp., exposure. Predicted molecular weights of proteins without tags (kDa): ARD 1–3: 21, ARD 4–6: 25; predicted molecular weights of oxomers without tags (kDa): FIH-IκBβ ARD 1–3: 61, FIH-IκBβ ARD 4–6: 65. Data are representative of three (B), one (C and E) and two (D) independent experiments.

We next aimed to identify the IκBβ amino acid targeted by FIH for oxomer formation. FIH is known to hydroxylate asparagine residues and the FIH-OTUB1 oxomer is formed through asparagine 22 (N22) of OTUB1.^4^^,^^26^ IκBα has so far been the best characterized FIH target protein outside the HIF pathway,^17^^,^^34^ it is hydroxylated by FIH on the two asparagine residues Asn-244 and Asn-210 and it is closely related to IκBβ.^17^ We therefore aimed to compare the IκBα and IκBβ structures to obtain insights into the potential part of IκBβ that might be targeted by FIH enzymatic activity. However, to the best of our knowledge, the protein structure of human IκBβ has not been experimentally solved to date. We therefore used AlphaFold to predict the structure of IκBβ and superimposed the obtained structure with the structure of IκBα.^35–37^ Figure 4A highlights the asparagine residues of IκBα that are hydroxylated by FIH and which are located within a loop between ARDs 2 and 3.^17^ Interestingly, one IκBβ asparagine residue (N266) was positioned in a comparable area of IκBβ as the hydroxylated asparagine residues in IκBα (Figure 4A). Although this indicates that N266 may be the targeted amino acid residue within IκBβ, we decided to point mutate all of the five IκBβ asparagine residues to alanine. In addition, one aspartate residue was also point mutated to alanine (D195A), as previous mass spectrometry analyses had shown its oxidation and because FIH also hydroxylates aspartate residues.^12^^,^^38^ AlphaFold-mediated structure prediction indicated that the introduced point mutations do not alter the structure of IκBβ (Figure S4A). Unexpectedly, none of the introduced IκBβ point mutations prevented FIH-IκBβ oxomer formation (Figure 4B). The higher molecular weight signal of the oxomer doublet showed some variability within its intensity (Figure 4B), but this was likely due to differences in the ectopic expression efficiency of the mutated IκBβ (Figure 4B). Thus, in contrast to the asparagine residue in the FIH-OTUB1 oxomer, FIH appears to utilize a different aa residue for oxomer formation with IκBβ.

Next, we analyzed whether IκBβ is hydroxylated by FIH via mass spectrometry (MS), because for OTUB1 the same asparagine residue that is involved in oxomer formation is also hydroxylated in monomeric OTUB1.^26^ We identified IκBß peptides containing four of the five asparagine residues. Despite detecting numerous oxidations on methionines, none of the asparagines were deemed to be hydroxylated (Supplemental Table S1). The one unidentified asparagine resides in the N-terminal region and was not accessible by standard MS workflows because the tryptic digest generated a peptide that was too large for MS identification. Nevertheless, it is highly likely that IκBß is not hydroxylated as none of the sequences surrounding any of the IκBß asparagines matches the well-characterized FIH consensus motif Lx(6)[VI]N.^39^ Therefore, we systematically screened for the IκBβ moiety that was involved in FIH-IκBβ oxomer formation. First, we assessed the N-(ARDs 1–3, aa residues 1–166) or C-terminal (ARDs 4–6, aa residues 167–356) parts of IκBβ (Figure 4C). According to AlphaFold predictions, these truncations largely maintained the corresponding protein structures (Figure S4B). While IκBβ ARDs 1–3 formed an oxomer with FIH with the predicted molecular weight, there was no oxomer formed with ARDs 4–6 (Figure 4C).

To identify the IκBβ residues involved in oxomer formation, vectors were constructed containing IκBβ ARD 1–3 with truncations of 10 aa increments from the C-terminal end (Figure 4D). Truncated IκBβ fragments containing the N-terminal 126 aa or less were no longer able to form an oxomer with FIH (Figure 4D). Next, IκBβs with consecutive deletions were generated in otherwise full-length IκBβ with overlapping steps of 5 aa, surrounding the identified area (Figure 4E). While deletion of IκBβ aa 127–136, 132–141 and 137–146 did not interfere with oxomer formation, the absence of IκBβ aa 117–126 and 122–131 abrogated oxomer formation (Figure 4E). Therefore, the IκBβ aa 122–126 (VAERR) region, common to both deletion constructs, is necessary for oxomer formation between FIH and IκBβ. These residues are located in the loop between the IκBβ ARDs 2 and 3 (Figure S4C and D). Of note, the IκBβ peptide containing VAERR was detected in the MS analyses, but no relevant amino acid residue was deemed to be hydroxylated within this peptide. Overall, the IκBβ VAERR sequence is necessary for FIH-IκBβ oxomer formation, but it remains unclear if any of the included amino acid residues is directly targeted by FIH enzymatic activity.

### FIH-IκBβ oxomer formation prevents IκBβ from binding the major NF-κB subunits p65 and c-Rel

**Figure 5.**
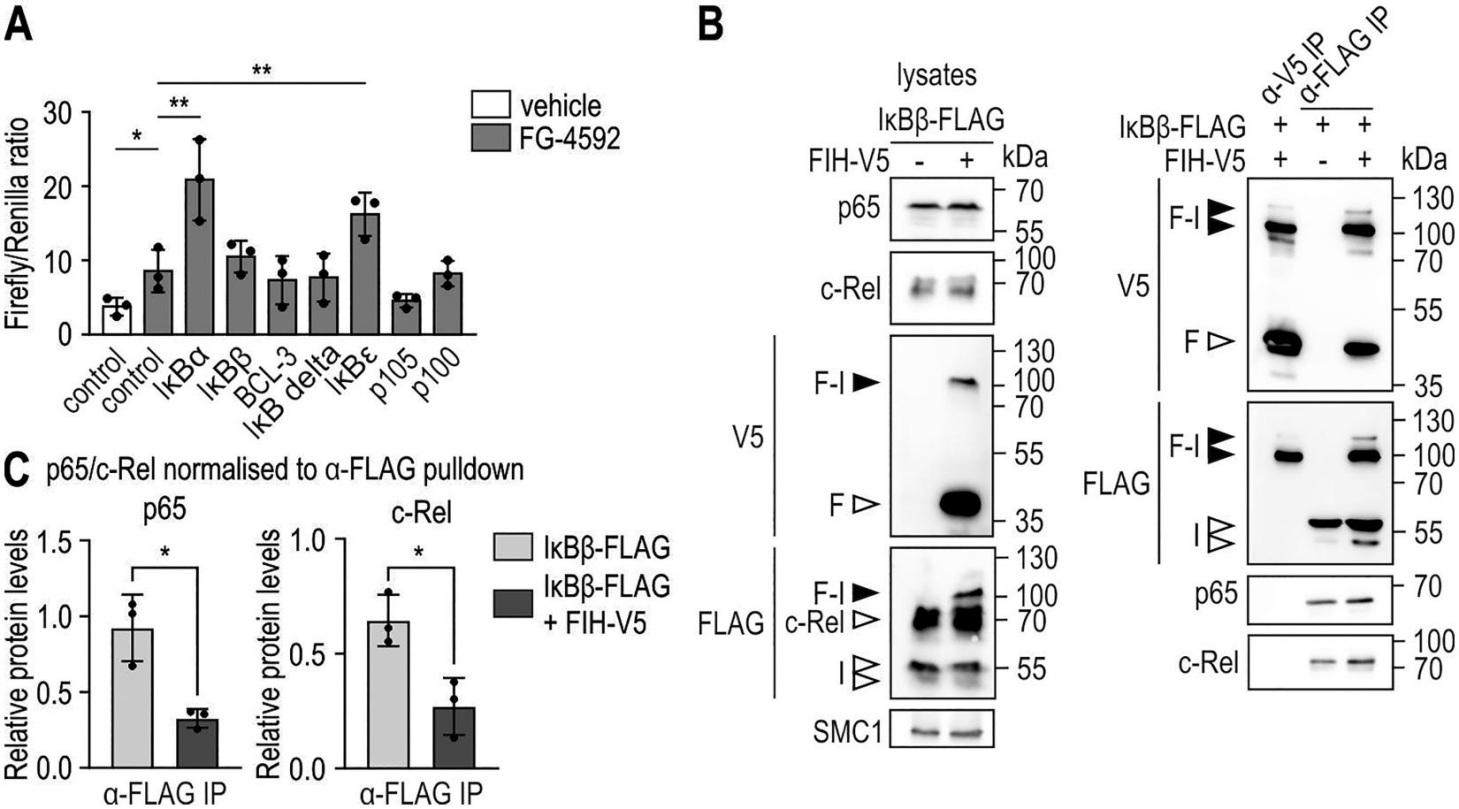
FIH-IκBβ oxomer formation prevents IκBβ from binding NF-κB subunits. (A) HEK293 cells were transiently cotransfected with a HIF-driven luciferase plasmid (pH3SVL) together with a constitutive *Renilla* luciferase plasmid (pRL-SV40) as well as plasmids expressing the indicated IκB protein family member 24 h prior to treatment. FG-4592 treatment was applied for 24 h in a concentration of 0.1 mM. Efficient transfection and FG-4592 treatment were determined by immunoblotting (Figure S5). Significance was assessed by two-way ANOVA with Tukey’s correction; *P < 0.05, **P < 0.01. (B) Immunoprecipitation (IP) analysis of the interaction of IκBβ and the FIH-IκBβ oxomer with the NF-κB subunits c-Rel and p65. (C) Quantification of the results shown in B. The p65 and c-Rel levels were normalized to the levels of total bait/FLAG pulldown (IκBβ or IκBβ + oxomer), respectively. Statistical analysis was performed by Student’s *t* test. *P < 0.05; F, FIH-V5; I, IκBβ-FLAG. Data are shown as mean ± SD. Data are representative of three independent experiments.

It has previously been suggested that IκBα can scavenge FIH from HIF-1α, reducing or preventing FIH-mediated hydroxylation of HIF-1α, and thus increasing HIF-1 transcriptional activity.^17^ Therefore, we aimed to investigate a potential effect of IκBβ on FIH activity through oxomer formation by employing a HIF-dependent firefly luciferase reporter gene assay.^40^^,^^41^ HEK293 cells were transfected with various IκB proteins for 24 h and treated with FG-4592 for an additional 24 h to specifically inhibit the PHDs to increase HIF activity without pharmacologically affecting FIH.^27^ FG-4592 treatment increased HIF-dependent firefly luciferase activity (Figure 5A). Ectopic expression of IκBα and IκBε significantly increased HIF activity likely by reducing FIH activity towards HIF (Figure 5A), as previously described.^17^^,^^22^ Interestingly, p105 had no additional effect compared to FG-4592 treatment alone (Figure 5A), although p105 is hydroxylated by FIH.^17^ No other IκB protein family member, including IκBβ, affected HIF activity. These results indicate that the effect of IκBα and IκBε towards HIF activity is selective and not a general effect of all FIH target proteins or all ARD-containing FIH substrates.

The main function of IκBβ is to bind to NF-κB transcription factor dimers, which sequesters them in the cytosol and regulates NF-κB-mediated gene expression.^20^^,^^21^ Thus, we investigated whether IκBβ retains its capability to bind NF-κB proteins within the oxomer. Specifically, the interaction between IκBβ and p65 as well as c-Rel was assessed, as these NF-κB subunits are most commonly bound by IκBβ.^42^ IκBβ-FLAG was ectopically expressed in HEK293 cells alone or in combination with FIH-V5, and immunoprecipitated using anti-V5 or anti-FLAG antibodies, respectively. The FLAG IP pulled down the FIH-IκBβ oxomer as well as monomeric IκBβ, whereas the V5 IP pulled down the oxomer as well as FIH but no monomeric IκBβ (Figure 5B), indicating that FIH was bound as homodimer to IκBβ as observed within the FIH-OTUB1 oxomer.^26^ p65 and c-Rel were both co-immunoprecipitated together with IκBβ-FLAG, but not in the FIH-V5 IP (Figure 5B and 5C), which contained IκBβ only as oxomer. These results suggest that oxomer formation of IκBβ with FIH prevents IκBβ from binding p65 and c-Rel and therefore removes IκBβ from the functionally relevant pool of IκB proteins for the regulation of NF-κB.

## Discussion

Over the last years, it has become apparent that a tight functional connection exists between hypoxia and inflammation.^1^^,^^8^^,^^11^^,^^43–46^ Tissue hypoxia is commonly observed in inflamed tissue areas and an inflammatory response can occur in hypoxic tissues.^9^^,^^46^ Furthermore, pharmacologic hydroxylase inhibitors demonstrated anti-inflammatory effects in numerous different inflammatory diseases in mice.^8–10^ Our understanding of the molecular mechanisms underlying this mutual interplay, however, is in its infancy. FIH may be part of the regulatory network connecting hypoxia and inflammation, as FIH alters IL-1β-induced NF-κB activity and is relevant for the inflammatory response in the gut *in vivo*.^12^^,^^14^^,^^15^ Nonetheless, the extent of contribution of FIH activity to inflammation as well as the molecular mechanisms that connect FIH and inflammation are unclear.

Previously, we reported that FIH forms an oxomer with OTUB1.^26^ Here, we demonstrate that FIH forms a similar oxomer specifically with IκBβ. FIH enzymatic activity was necessary for the FIH-IκBβ complex formation, which was prevented by pan-hydroxylase inhibitors and FIH mutations. The oxomer was formed in several different cell lines and by endogenous proteins. The endogenous oxomer was observed at relatively low levels compared to the IκBβ monomer. However, this assessment was performed in HEK293 cells and may differ in other cell lines. Interestingly, we observed (with ectopically expressed proteins) that the oxomer level relative to monomeric IκBβ is higher in MCF7 than in HEK293, Hep3B and HepG2 cells, indicating that there might be cell types in which oxomer formation is favored. Moreover, following a pro-inflammatory stimulus, IκBβ is degraded and re-expressed.^47^^,^^48^ We have previously reported that oxomer formation occurs co-translationally.^26^ Thus, following a pro-inflammatory stimulus, (endogenous) oxomer formation may be increased due to the strong induction of IκBβ expression and translation. This will be analyzed in the future.

Interestingly, IκBβ was the only protein of the IκB protein family that was targeted by FIH for oxomer formation, although all members contain ARDs which are prominent FIH substrates.^17^^,^^22^^,^^23^ Within our initial screen for FIH target proteins for oxomer formation, IκBβ was the only identified ARD-containing protein.^26^ Together, these results demonstrate that FIH-mediated oxomer formation is highly selective and indicate that ARDs are not a defining characteristic of substrate proteins for FIH-dependent oxomer formation.

The FIH-mediated FIH-IκBβ oxomer formation demonstrated a higher hypoxia sensitivity than the PHD-mediated stabilization of HIF-1α. Moreover, this sensitivity was even higher than the sensitivity of FIH-mediated HIF-1α hydroxylation, which is known to be less susceptible to hypoxia than PHD-mediated hydroxylation.^49^ These results are remarkable and comparable to our previous findings regarding the hypoxia sensitivity of the FIH-OTUB1 oxomer formation.^26^ This further strengthens the hypothesis that oxomer formation serves as an alternative cellular mechanism to fine-tune the adaptation to hypoxia. Moreover, it indicates that the hypoxia sensitivity of oxomer formation is largely defined by yet unknown characteristics of FIH and less by its protein substrates. In addition, the FIH-IκBβ oxomer was very stable and only started to noticeably decline 24 h after the onset of hypoxia. These results are consistent with the FIH-OTUB1 oxomer and support the conclusion that FIH-dependent oxomers may serve for signaling long-term rather than short-term fluctuations in local oxygen availability.^26^

As previously observed for the FIH-OTUB1 oxomer,^27^ the FIH-selective inhibitor DM-NOFD failed to prevent FIH-IκBβ oxomer formation. Of note, both oxomers showed a higher hypoxia sensitivity than FIH-mediated HIF-α asparagine hydroxylation,^26^ indicating that the sensitivity of FIH for co-substrate binding depends on the protein substrate. This may also extend to co-substrate mimetics, such as DM-NOFD. To test this hypothesis, it would be necessary to assess the DM-NOFD sensitivity of FIH-dependent HIF-α asparagine hydroxylation and oxomer formation within the same lysates. However, a reliable antibody detecting HIF-α asparagine hydroxylation is currently not commercially available.

The connection between FIH and IκBβ in the FIH-IκBβ oxomer resisted high concentrations of reducing agents and boiling, suggesting a likely covalent bond, and indicating that the oxomer is formed independently of cysteine residues. FIH hydroxylates asparagine residues^4^ and the FIH-OTUB1 oxomer is formed through asparagine 22 (N22) of OTUB1. However, FIH is known to be promiscuous^16^ and hydroxylates aspartate,^38^ histidine,^50^ and tryptophan residues as well.^51^ Interestingly, we observed that point mutations of each asparagine within IκBβ did not interfere with oxomer formation, demonstrating that the FIH-IκBβ oxomer was not formed via an IκBβ asparagine residue. IκBβ-targeted MS analysis did not identify any potential hydroxylation of IκBβ, indicating that the previously identified oxidation of D195 likely occurred by random reaction with oxygen during sample preparation and not by enzymatic activity.^12^ In addition, IP of FIH pulled down the FIH-IκBβ oxomer, but not the IκBβ monomer. Together, these results strongly indicate that IκBβ is not hydroxylated by FIH. Previously, in an in vitro decarboxylation assay using purified FIH and IκBβ, no FIH-dependent activity toward IκBβ was detected,^17^ further supporting that IκBβ is not a hydroxylation substrate of FIH. Nonetheless, the results obtained by the in vitro assay are not contradicting our observation of the FIH-IκBβ oxomer formation, because such oxomers are likely cotranslationally formed.^26^ Assuming that also the FIH-IκBβ oxomer is formed cotranslationally, such a formation would not be detected by an assay that utilizes fully folded proteins.

Analyzing possible functional effects of the FIH-IκBβ oxomer, the FIH-dependent regulation of HIF transactivation activity appeared to be unaffected in a HIF-dependent reporter gene assay. In the same experiment, ectopic expression of IκBα and IκBε increased HIF-dependent firefly luciferase activity, which is in agreement with a previous report that IκBα can inhibit FIH activity toward HIF-1α.^52^ To our knowledge, this is the first report that also IκBε – but no other IκB protein family member – affects HIF activity.

The main function of IκBβ is to bind to NF-κB transcription factor dimers (mostly containing c-Rel and p65) and to retain them in the cytosol, preventing gene expression enhancement by these specific NF-κB dimers.^42^ It is therefore of interest that IκBβ complexed with FIH as oxomer did not retain its ability to interact with the NF-κB subunits p65 and c-Rel. Consequently, oxomer formation may increase NF-κB-dependent transcription by preventing p65 and c-Rel binding to IκBβ. Such a regulation would be specific to NF-κB dimers involving p65 and/or c-Rel and would thus not necessarily affect the overall NF-κB-dependent gene expression, as 13 different NF-κB dimers have (so far) been demonstrated to exist.^53^ Further investigations will be necessary to determine the extent of the contribution of FIH-IκBβ oxomer formation to the regulation of pro-inflammatory gene expression via inhibition of IκBβ binding to NF-κB dimers.

Recently, it has been reported that FIH expression is increased in inflamed renal tissue during chronic kidney disease (CKD).^54^ It would therefore be of interest to test for the existence and potential regulation of the FIH-IκBβ oxomer in healthy and diseased renal tissues, as an enhanced FIH-IκBβ oxomer formation may contribute to increased inflammation. However, it is currently unclear to which extent oxomers exist in healthy or diseased tissues.

In summary, FIH forms a previously unknown oxomer with IκBβ in a likely covalent manner and with high hypoxia sensitivity. FIH-mediated sequestration of IκBβ within an oxomer does not affect the total FIH enzymatic activity present within a cell, but it prevents the complexed IκBβ from inhibiting specific NF-κB dimers. Therefore, the FIH-IκBβ oxomer formation and/or its hypoxic inhibition may contribute to the interplay between hypoxia/FIH and inflammation.

## Materials and Methods

### Cell culture and transient transfection

Human HEK293 (embryonic kidney), HepG2 and Hep3B (hepatocellular carcinoma), and MCF7 (breast adenocarcinoma) cell lines were cultivated in high glucose Dulbecco’s modified Eagle’s medium (Sigma-Aldrich, USA) with supplementation of 100 U/mL penicillin and 100 μg/mL streptomycin (Sigma-Aldrich) as well as 10% heat-inactivated fetal calf serum (Gibco, USA). Transient transfection of plasmids was performed with lipofectamine 2000 reagent according to the manufacturers’ protocol (Invitrogen, USA).

### RNA isolation and RT-qPCR

RNA was extracted by the guanidinium thiocyanate-acid phenol-chloroform method as described earlier.^55^ Complementary DNA (cDNA) was obtained by reverse transcription (RT) with AffinityScript transcriptase (Agilent, Santa Clara, CA, USA) and quantitated using the SYBR Green qPCR reagent kit (Kapa Biosystems, London, UK) and a MX3000P light cycler (Agilent) using standard curves. Transcript levels were normalized to the levels of human ribosomal protein L28 mRNA. Primer sequences are listed in Supplemental Table S2.

### Cell treatment

Cells were treated with the following compounds dissolved in dimethylsulfoxide (DMSO, Sigma-Aldrich)^56^ and the final concentrations indicated: dimethyloxalylglycine (DMOG; Frontier Scientific, Logan, UT, USA; 1 mM), FG-4592 (roxadustat; Selleckchem, Houston, TX, USA; 0.1 mM) and dimethyl N-oxalyl-D-phenylalanine (DM-NOFD; Sigma-Aldrich; 1 mM). Desferrioxamine (DFX; Sigma-Aldrich; 0.1 mM) was dissolved in bidistilled water. MG132 (Sigma-Aldrich; 10 μM) was dissolved in ethanol (Sigma-Aldrich). To test the sensitivity of the oxomer against reducing agents, cell lysates were treated with dithiothreitol (DTT; Agilent Technologies) or β-mercaptoethanol (Sigma-Aldrich).

Incubations in hypoxia were performed using the InvivO_2_ 400 humidified cell culture workstation (Baker Ruskinn, Bridgend, South Wales, UK) operated with 0.2% O_2_ or 1% O_2_, 5% CO_2_ at 37 °C as previously described, or in humidified oxygen-regulated cell culture incubators (Binder, Tuttlingen, Germany) operated with 2–8% O_2_ and 5% CO_2_ at 37 °C.^57^ ”normoxia” refers to the air oxygen level in the gas phase within a humidified cell culture incubator at 500 m altitude (18.5% O_2_).^58^

### Reporter gene assays

HEK293 cells were cotransfected with pH3SVL, encoding the firefly (*Photinus pyralis*) luciferase reporter gene under the control of a SV40 promoter and concatamerized transferrin-derived hypoxia response elements (HREs) (total of six HIF binding sites) and pRL-SV40, encoding a constitutively expressed *Renilla* (*Renilla reniformis*) luciferase reporter construct driven by the SV40 promoter, in combination with a member of the IκB protein family (IκBα, IκBβ, Bcl-3, IκBδ, IκBε, p105, p100) or empty vector.^40^^,^^41^ Twenty-four hours after transfection, cells were treated with 0.1 mM prolyl hydroxylase inhibitor FG-4592 (roxadustat) or vehicle (DMSO) for 24 h. Dual-Luciferase Reporter Assay was used to determine firefly and *Renilla* luciferase bioluminescence according to the manufacturers’ instruction (Promega, Madison, Wisconsin, USA). In brief, cells were washed with PBS and lysed with Passive Lysis Buffer (Promega) for 10 min at room temperature. Lysates were mixed with equal volumes of Luciferase Assay Reagent II and luminescence was measured by a microplate luminometer (Berthold Technologies, Bad Wildbach, Germany). Subsequently, freshly combined Stop & Glo reagent (Promega) was added and the activity of *Renilla* luciferase was determined.

### Protein structure prediction

Predictions of protein structures were created with ColabFold using the default settings, which resulted in five structure predictions, ranked by Predicted Aligned Error (PAE).^59^ Rank 1 model was used for further analysis and representation (https://colab.research.google.com/github/sokrypton/ColabFold/blob/main/AlphaFold2.ipynb).

### Statistical analysis

For the analysis of the significance of difference between two data points, Student’s *t* test was applied. For comparison of more than two data points, one-way or two-way ANOVA followed by Tukey’s post-test was applied. *P* values < 0.05 were considered statistically significant. The sample sizes for each experiment have been indicated in the corresponding figure legends.

## Supplementary Material

Supplemental Material

## Data Availability

All underlying raw data have been made available on https://dataverse.harvard.edu/. The data has obtained the DOI 10.7910/DVN/XYXNPA and is accessible under https://dataverse.harvard.edu/privateurl.xhtml?token=29762520-1072-418d-873a-5a110caa04cf. The mass spectrometry proteomics data have been deposited to the ProteomeXchange Consortium via the PRIDE^60^ partner repository with the dataset identifier PXD047442.
