# Supplementary material for: Selective Hypoxia-Sensitive Oxomer Formation by FIH Prevents Binding of the NF-κB Inhibitor IκBβ to NF-κB Subunits
Source: Mol Cell Biol. 2024 Apr 22;44(4):138–48. doi: 10.1080/10985549.2024.2338727 (PMC11110689; doi:10.1080/10985549.2024.2338727)
Supplement: Supplemental Material [file TMCB_A_2338727_SM3628.zip › Supplemental Material.docx]

SUPPLEMENTAL MATERIAL

Selective hypoxia-sensitive oxomer formation by FIH prevents binding of the NF-κB inhibitor IκBβ to NF-κB subunits

Yulia L. Volkova^a^, Agnieszka E. Jucht^a^, Nina Oechsler^b^, Roopesh Krishnankutty^c^, Alex von Kriegsheim^c^, Roland H. Wenger^a^, Carsten C. Scholz^a,b^

^a^Institute of Physiology, University of Zurich, Zurich, Switzerland

^b^Institute of Physiology, University Medicine Greifswald, Greifswald, Germany

^c^Institute of Genetics and Cancer, University of Edinburgh, United Kingdom

## SUPPLEMENTAL MATERIALS AND METHODS

Table S2. Primer sequences.

| **Primer name** | | **Primer sequence** |
| --- | --- | --- |
| **Primers for site-directed mutagenesis of human IκBβ (introduced nucleotide(s) highlighted in bold)** | | |
| N91A (a271g, a272c) | forward | 5'-agtacatggacctgcag**gc**tgacctaggccagacag-3' |
|  | reverse | 5'-ctgtctggcctaggtca**gc**ctgcaggtccatgtact-3' |
| N173A (a517g, a518c) | forward | 5'-cgtactcccgacacc**gc**ccatacccctgtcgc-3' |
|  | reverse | 5'-gcgacaggggtatgg**gc**ggtgtcgggagtacg-3' |
| D195A (a584c) | forward | 5'-ggagagtgaggagg**c**ctggaagctgcagc-3' |
|  | reverse | 5'-gctgcagcttccag**g**cctcctcactctcc-3' |
| N204A (a610g, a611c) | forward | 5'-cagctggaggctgaa**gc**ctacgagggccacac-3' |
|  | reverse | 5'-gtgtggccctcgtag**gc**ttcagcctccagctg-3' |
| N266A (a796g, a797c) | forward | 5'-agggcaggcgcg**gc**ccctgctgcccg-3' |
|  | reverse | 5'-cgggcagcaggg**gc**cgcgcctgccct-3' |
| N286A (a856g, a857c) | forward | 5'-catgctccggccc**gc**ccccatcctcgcc-3' |
|  | reverse | 5'-ggcgaggatgggg**gc**gggccggagcatg-3' |
| **Primers for partial protein deletions of human IκBβ (underscored nucleotides represent the sequence parts located after the introduced deletion, overlapping parts of the primers are highlighted in bold)** | | |
| Deletion 117-126 | forward | 5'-ccacggtggagaagctgt**acgcagcaggccacac**-3' |
|  | reverse | 5'-caggccaggtgcagcgcc**gtgtggcctgctgcgt**-3' |
| Deletion 122-131 | forward | 5'-tgtacgcagcaggcgccg**ggctgtgtcacctggc**-3' |
|  | reverse | 5'-tgtgcccccacacggcag**gccaggtgacacagcc**-3' |
| Deletion 127-136 | forward | 5'-ccgggctgtgtgtggcgg**agcgtagggtgggggc**-3' |
|  | reverse | 5'-ggcacgggcacaggcgtgt**gcccccaccctacgct**-3' |
| Deletion 132-141 | forward | 5'-cggagcgtaggggccaca**cggcgctgtgtgcccg**-3' |
|  | reverse | 5'-cggggctgaagcagggca**cgggcacacagcgccg**-3' |
| Deletion 137-146 | forward | 5'-acacggcgctgcacctgg**cctgccgtcttcagcc**-3' |
|  | reverse | 5'-ctggggcgccgggggcgg**ggctgaagacggcagg**-3' |
| **Primers for site-directed mutagenesis of human FIH (introduced nucleotide(s) highlighted in bold):** | | |
| L340R (t1018a, t1019g) | forward | 5'-gtggggcccttg**ag**gaacacaatgatcaagggc-3' |
|  | reverse | 5'-cttgatcattgtgttc**ct**caagggccccacctc-3' |

| **Primers for human IκBβ truncations (the sequence parts overlapping with IκBβ are highlighted in bold). Primers were designed according to the Gateway Technology Guidelines for attB PCR Primers.** | | |
| --- | --- | --- |
| ARD1-3 | forward | 5'-ggggacaagtttgtacaaaaaagcaggcttc**atggctggggtcgcgtgcttggg**-3' |
|  | reverse | 5'-ggggaccactttgtacaagaaagctgggtc**agggccctgagcgaggtaggtgtcg**-3' |
| ARD4-6 | forward | 5'-ggggacaagtttgtacaaaaaagcaggcttcatg**gaccgtactcccgacaccaac**-3' |
|  | reverse | 5'-ggggaccactttgtacaagaaagctgggtc**gcacacggggcgggggtc**-3' |
| ARD1 | forward | the same as ARD1-3 forward |
|  | reverse | 5'-ggggaccactttgtacaagaaagctgggtc**gtccatgtactcagtgccggccga**-3' |
| IκBβ  1-156 | forward | the same as ARD1-3 forward |
|  | reverse | 5'-ggggaccactttgtacaagaaagctgggtc**ttccctggggcgccgggggcgggg**-3' |
| IκBβ  1-146 | forward | the same as ARD1-3 forward |
|  | reverse | 5'-ggggaccactttgtacaagaaagctgggtc**cagggcacgggcacaggcgtgtgc**-3' |
| IκBβ  1-136 | forward | the same as ARD1-3 forward |
|  | reverse | 5'-ggggaccactttgtacaagaaagctgggtc**acggcaggccaggtgcagcgccgt**-3' |
| IκBβ  1-126 | forward | the same as ARD1-3 forward |
|  | reverse | 5'-ggggaccactttgtacaagaaagctgggtc**cctacgctccgccacacacagccc**-3' |
| IκBβ  1-116 | forward | the same as ARD1-3 forward |
|  | reverse | 5'-ggggaccactttgtacaagaaagctgggtc**tgctgcgtacagcttctccaccgt**-3' |
| IκBβ  1-106 | forward | the same as ARD1-3 forward |
|  | reverse | 5'-ggggaccactttgtacaagaaagctgggtc**ctcccccaggatggctgccaggtg**-3' |
| IκBβ  1-96 | forward | the same as ARD1-3 forward |
|  | reverse | 5'-ggggaccactttgtacaagaaagctgggtc**tgtctggcctaggtcattctgcag**-3' |
| **Primers for qPCR** | | |
| FIH | forward | 5'-cagttcgagtgcctctaccc-3' |
|  | reverse | 5'-atggccactttctgatgagc-3' |
| L28 | forward | 5'-gcaattccttccgctacaac-3' |
|  | reverse | 5'-tgttcttgcggatcatgtgt-3' |

## Immunoprecipitation

For endogenous FIH immunoprecipitation (IP), cells were lysed with 1% Triton X-100 in 20 mM Tris-HCl (pH 7.5), 150 mM NaCl, 1 mM MgCl_2_, and 100 μL of protein G-sepharose beads (GE Healthcare), coupled with anti-FIH (Novus Biologicals, NBP1-30333) or anti-β-actin (Sigma-Aldrich; A5441) antibodies, were added. Following overnight incubation at 4°C on a vertical rotator, samples were centrifuged at 27 × g for 30 s, the supernatant was removed, samples were washed twice with 300 μL of lysis buffer and twice with 300 μL of washing buffer (20 mM Tris-HCl pH 7.5, 150 mM NaCl, 1 mM MgCl_2_). The samples were boiled in 30 μL of non-reducing sample loading buffer (50 mM Tris-HCl pH 6.8, 6% glycerol, 2% SDS, 0.01% bromophenol blue) for 5 min. Following centrifugation at 27 × g for 30 s, the supernatant was transferred to new tubes. 10 mM DTT was added and the samples were boiled for 5 minutes. Sample analyses were performed by immunoblotting.

For precipitation of V5 and FLAG-tagged proteins, IP was performed as described above using anti-FLAG M2 antibody-coupled agarose beads (Sigma-Aldrich) or anti-V5 agarose affinity gel (Sigma-Aldrich). Cell lysates were incubated with the corresponding beads for 1 h at 4°C on a vertical rotator.

## Plasmids

Plasmids encoding FIH-V5 and FLAG-OTUB1 have previously been described ^1^. The vectors encoding FIH H199A-V5 and FLAG-tagged UBA1 were kind gifts from Daniel J. Peet (Department of Molecular and Biomedical Science, University of Adelaide, Australia) and Angelos Constantinou (Institut de Génétique Humaine, Centre National de la Recherche Scientifique, Université de Montpellier, France), respectively.^2, 3^ The vectors encoding V5-IκBβ and IκBβ-FLAG were produced by recombining the IκBβ coding sequence from the pDONR223 plasmid (pDONR223_NFKBIB_WT, Addgene, #82219, <https://www.addgene.org/>) with destination vectors containing a V5 tag (pcDNA3.1/nV5-DEST) or a triple C-terminal FLAG tag (pCSF107mT-GATEWAY-3'-FLAG, Addgene, #67619) using LR clonase according to the manufacturer´s instructions (Gateway cloning technology; Life Technologies, USA). Plasmids encoding IκB protein family members Bcl-3 (HsCD00862169), p100 (HsCD00860317), p105 (HsCD00079944), and IκBε (HsCD00829409) were in the pDONR221 vector; IκBδ (HsCD00956199) was in pANT7_cGST (DNASU Plasmid Repository, <https://dnasu.org/DNASU>). The coding sequence of IκBα was introduced into the Gateway cloning system by ligating the sequence from the pCMV4-3 HA/IkB-alpha vector (Addgene, Plasmid #21985) to pENTR4. All listed IκB proteins were recombined into the destination vector pCSF107mT-GATEWAY-3'-FLAG.

## Truncations, partial protein deletions and site-directed mutagenesis

To obtain truncated IκBβ, full-length IκBβ was used as a template for the amplification of the desired IκBβ cDNA parts using specifically designed primers. The ARDs 1-3 of IκBβ contain the N-terminal amino acids (aa) 1 to 166 and ARDs 4-6 contain the C-terminal aa 167 to 356, the end of full-length IκBβ, respectively. PCR products were purified by 2% agarose gel electrophoresis and extracted using the NucleoSpin Gel and PCR Clean‑up kit (Macherey-Nagel, Germany). Truncated IκBβ was recombined into pDONR221 (Addgene, #2394) using the Gateway Cloning technology and later inserted to pCSF107mT-GATEWAY-3'-FLAG via recombination. The FIH L340R plasmid was created using the FIH-V5 plasmid as a templated and the Quickchange II XL Site-Directed Mutagenesis kit according to the manufacturer's instructions (Agilent Technologies). The IκBβ point mutations N91A, N173A, D195A, N204A, N266A, N286A as well as the deletions of regions containing aa 117-126, 122-131, 127-136, 132-141 or 137-146 were introduced with the Quickchange II XL Site-Directed Mutagenesis kit using IκBβ-FLAG as template. All truncations, point mutations and deletions were confirmed by DNA sequencing (Microsynth AG, Switzerland). Primers are listed in the Supplemental table S1.

## Gene deletion

A CRISPR/Cas-derived HEK293 FIH knockout (KO) cell pool was kindly provided by Anja Bremm (Institute of Biochemistry II, Goethe-University, Frankfurt am Main, Germany).^4^ Cell cloning was performed by limiting dilution and two separate FIH KO HEK293 cell clones were chosen following immunoblot and RT-qPCR analyses (Fig. S1).

## Immunoblotting

Cells were lysed with 1% NP-40 in 150 mM NaCl, 25 mM Tris-HCl (pH 8.0), 1 mM EDTA, and freshly added protease inhibitor cocktail (Sigma-Aldrich), 1 mM PMSF and 1 mM Na_3_VO_4_. Protein concentrations were determined using the BCA assay (Thermo Fisher Scientific, Waltham, MA, USA). Samples were mixed with sample loading buffer (250 mM Tris-HCl pH 6.8, 30% glycerol, 858 mM β-mercaptoethanol, 10% SDS, 0.05% bromophenol blue) in a 4:1 ratio, separated by SDS-PAGE and electro-transferred to nitrocellulose membranes (Cytiva, USA). Primary antibodies used for detection are as follows: anti-V5 (Invitrogen; R960-025; dilution 1:5000), anti-FLAG (Sigma-Aldrich; F3165; 1:5000), anti-α-tubulin (Cell Signaling; 2144; 1:1000), anti-FIH (Novus Biologicals, NBP1-30333; 1:5000), anti-SMC1 (Abcam, Cambridge, UK; 9262; 1:1000), anti-HIF-1α (BD Biosciences, San Jose, CA, USA; 610959; 1:500), anti-IκBβ (Cell Signaling; 8635; 1:1000), anti-β-actin (Sigma-Aldrich; A5441; 1:10000), anti-p65 (Santa Cruz Biotechnology; sc-8008; 1:1000) and anti-c-Rel (Cell Signaling; 4727; 1:500). Secondary antibodies: horseradish peroxidase-coupled goat anti-mouse, goat anti-rabbit and rat anti-mouse (Thermo Fisher Scientific, 31430, 31460; Abcam, 131368, for the detection of immunoprecipitated samples). Horseradish peroxidase activity was detected with SuperSignal enhanced chemiluminescence substrate (Thermo Fisher Scientific) and chemiluminescence was recorded using a CCD camera (Fusion FX7 Vilber, Germany). ImageJ was used for quantification as described previously.^5^ Values were normalized to the respective loading controls.

## Mass spectrometry-based analysis of the IκBβ hydroxylation status

The desalted and lyophilized peptides were resuspended in 0.1% TFA and subjected to mass spectrometric analysis by reversed-phase nano–liquid chromatography–tandem mass spectrometry (LC-MS/MS). Mass spectrometry: 5 µl of the resuspended peptides was analyzed by reversed-phase nano–LC-MS/MS using a nano-Ultimate 3000 LC system and a Lumos Fusion mass spectrometer (Thermo Fisher Scientific). Flow rates were 400 nl/min. Peptides were loaded onto a self-packed analytical column (uChrom 1.6, 0.075 mm by 25 cm) using a 67-min gradient buffer A (2% acetonitrile, 0.5% acetic acid) and buffer B (80% acetonitrile, 0.5% acetic acid); 0 to 16 min: 2% buffer B, 16 to 56 min: 3 to 35% buffer B, 56 to 62 min: 99% buffer B; 62 to 67 min 2% buffer B. Full-scan spectra recording in the Orbitrap was in the range of m/z 350 to m/z 1,400 (resolution: 240,000; AGC: 7.5e5 ions). MS2 was performed in the ion trap, with an isolation window of 0.7, an AGC of 2e4, an HCD collision energy of 28, rapid scan rate, a scan range of 145 to 1450 m/z, 50-ms maximum injection time, and an overall cycle time of 1 s.

Database search: The mass spectrometry raw data were analyzed by the FragPipe software package^6^ using the preselected conditions for analysis (specific proteases, two missed cleavages, and seven amino acids minimum length, recalibr). Protease was set to trypsin. Carbamylation (C) was selected as fixed modification. Variable modifications were N-terminal acetylation (protein) and oxidation (MNPH). False discovery rate was set to 0.01. MS/MS spectra were searched against the human UniProt database with an initial mass accuracy of 20 parts per million (ppm; for MS) 0.5 Da (MS/MS).

## SUPPLEMENTAL FIGURES


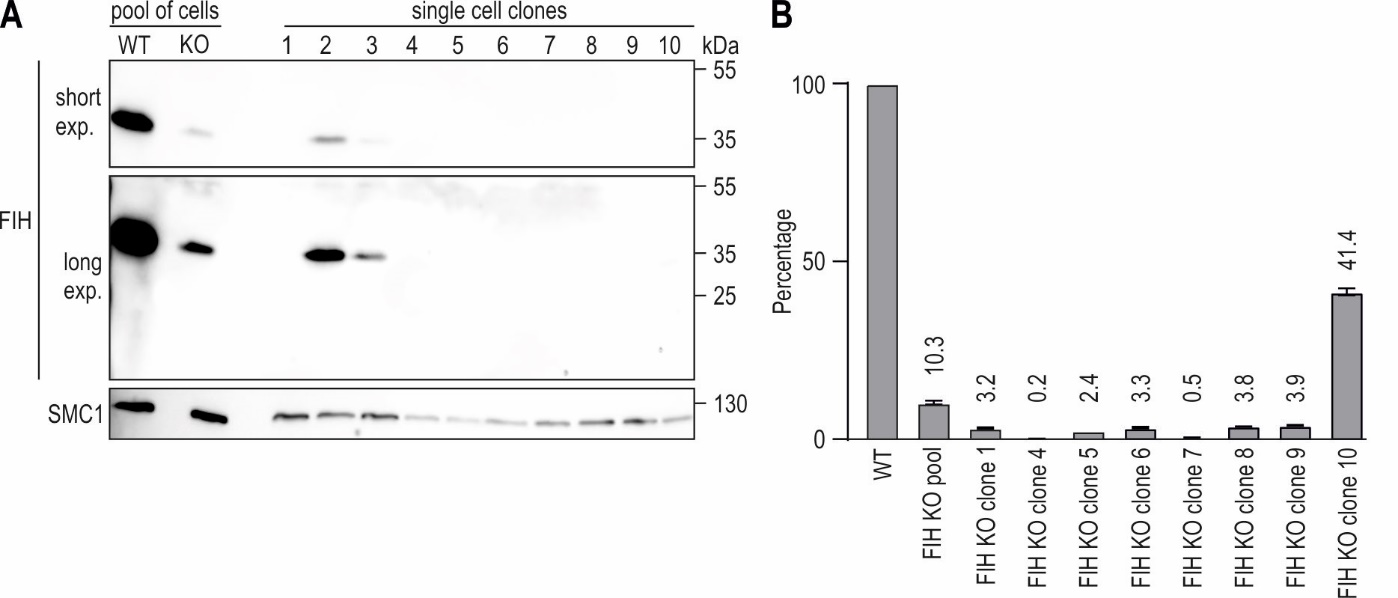


Figure S1. Cloning of HEK293 FIH knockout cell lines. (A) Immunoblot analysis of HEK293 wild type (WT) cells, a pool of HEK293 cells modified using CRIPSR/Cas technology to obtain FIH knockout (KO)s and single cell clones derived from the FIH KO pool. (B) Relative FIH mRNA levels obtained by RT-qPCR analyses of the same cells as shown in (A). Numbers indicate the calculated mRNA percentage relative to WT levels. Data are shown as mean ± SEM. Data are representative of one (A) and two (B) independent experiments. Clones 8 and 9 were chosen for further experiments.


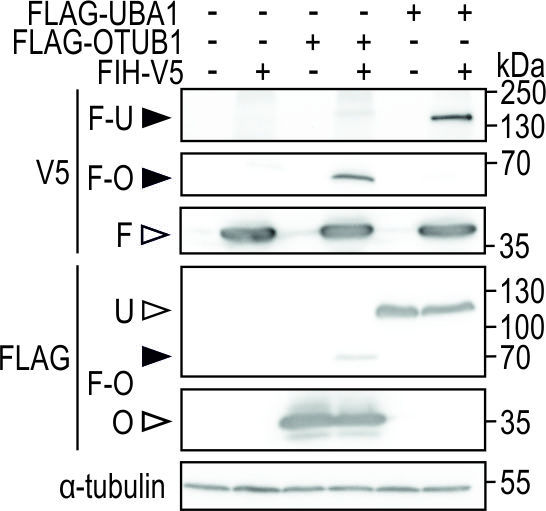


Figure S2. Formation of an oxomer-like complex between FIH and UBA1. Immunoblot analysis of potential oxomer formation between ectopically expressed FIH-V5 and FLAG-UBA1 in HEK293 cells, using the previously reported FIH-OTUB1 oxomer formation as control ^1^. F, FIH-V5; U, FLAG-UBA1; O, FLAG-OTUB1; F-O, FIH-OTUB1 oxomer; F-U, putative FIH-UBA1 oxomer.


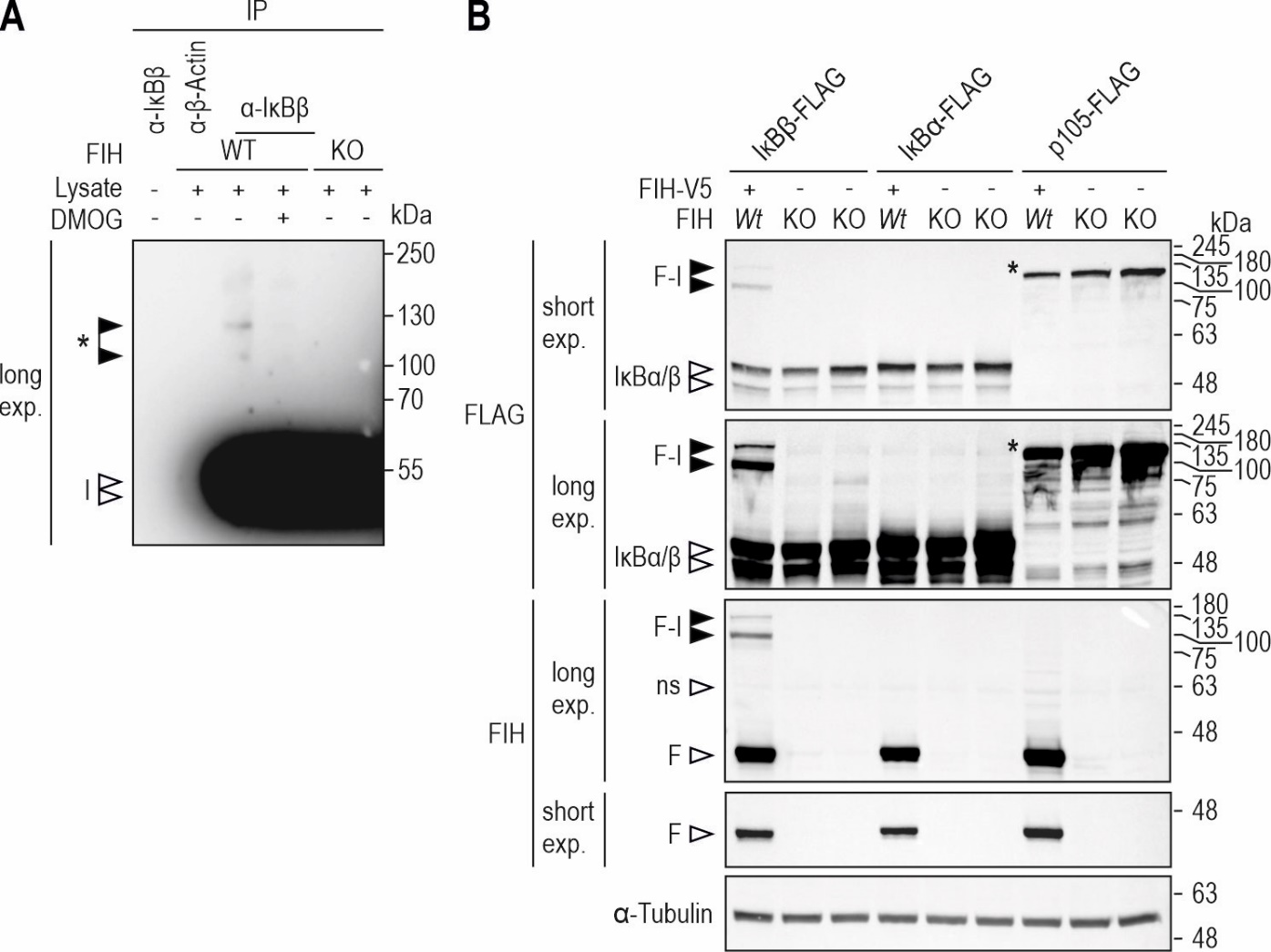


Figure S3. Selective oxomer formation between FIH and IκBβ. (A) Larger image section of the long exposure IκBβ immunoblot shown in Figure 1C, including the detection of monomeric IκBβ. (B) Immunoblot analysis of oxomer formation between FIH-V5 and the selected members of the IκB protein family following transient transfection of HEK293 wildtype or FIH knockout cells (two separate clones) with the indicated vectors for ectopic expression. The three immunoblot signals on the right of “*” are signals derived from ectopically expressed p105. Data are representative of two (A) and three (B) independent experiments. F, FIH-V5; I, V5-IκBβ; F-I, FIH-IκBβ oxomer; exp, exposure; ns, non-specific.


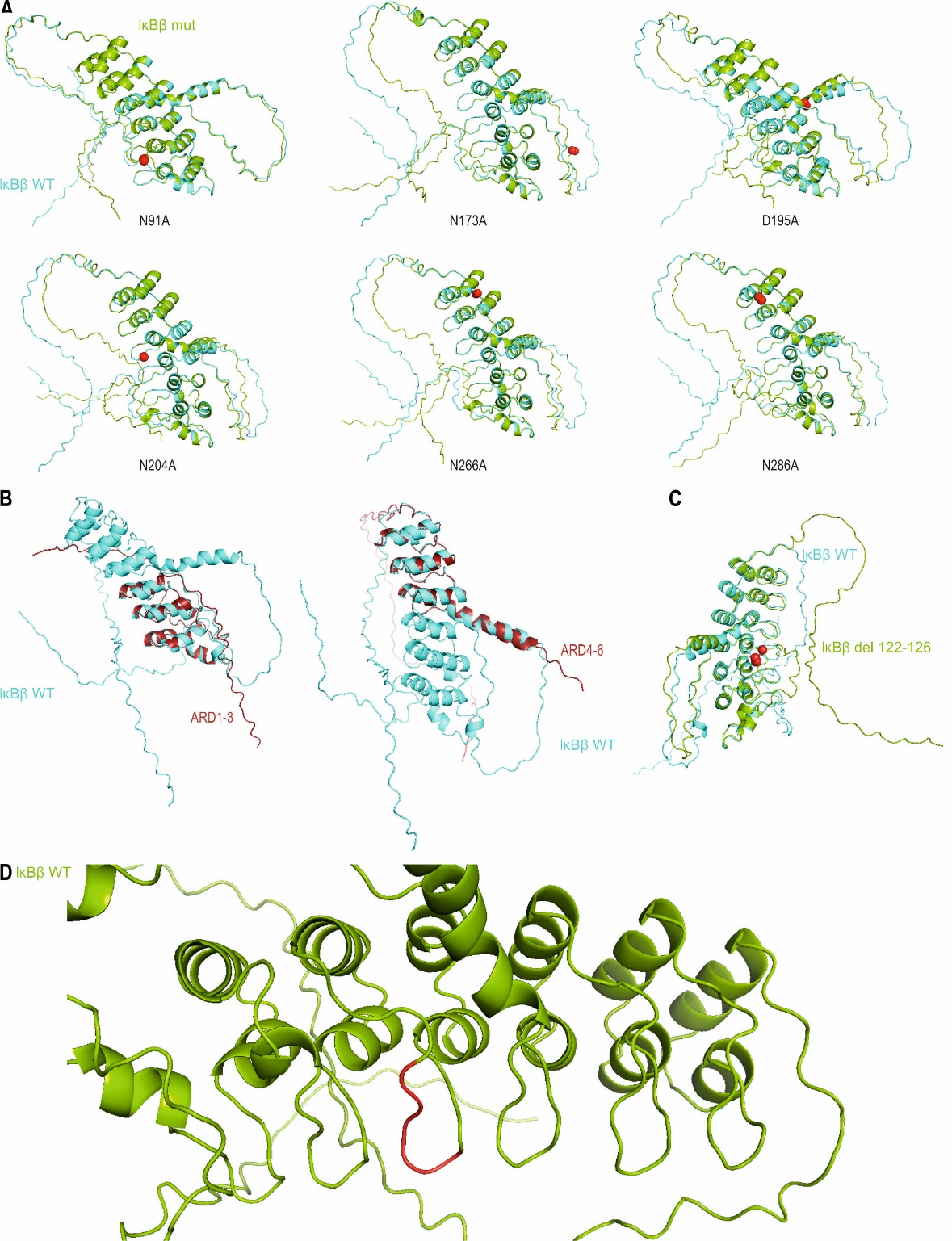


Figure S4. Structure predictions of IκBβ. (A) Structural comparison of predicted IκBβ WT (cyan; obtained from AlphaFold Protein Structure Database; identifier AF-Q15653-F1) and IκBβ containing the highlighted point mutations (green; mutated amino acids are highlighted in red; structure predicted by ColabFold (AlphaFold2 using MMseqs2).^7^ (B) Comparison of structure predictions from IκBβ WT (cyan) and IκBβ containing ankyrin repeat domains 1 to 3 (aa 1-166, red; ColabFold) or ankyrin repeat domains 4 to 6 (aa 167-356, red; ColabFold). (C) Structure predictions of IκBβ WT (cyan) and IκBβ containing a deletion of the amino acids 122-126 (green; ColabFold). Amino acids aimed for deletion are depicted in red on the WT structure. (D) Location of amino acids 122-126 (in red) with IκBβ WT.


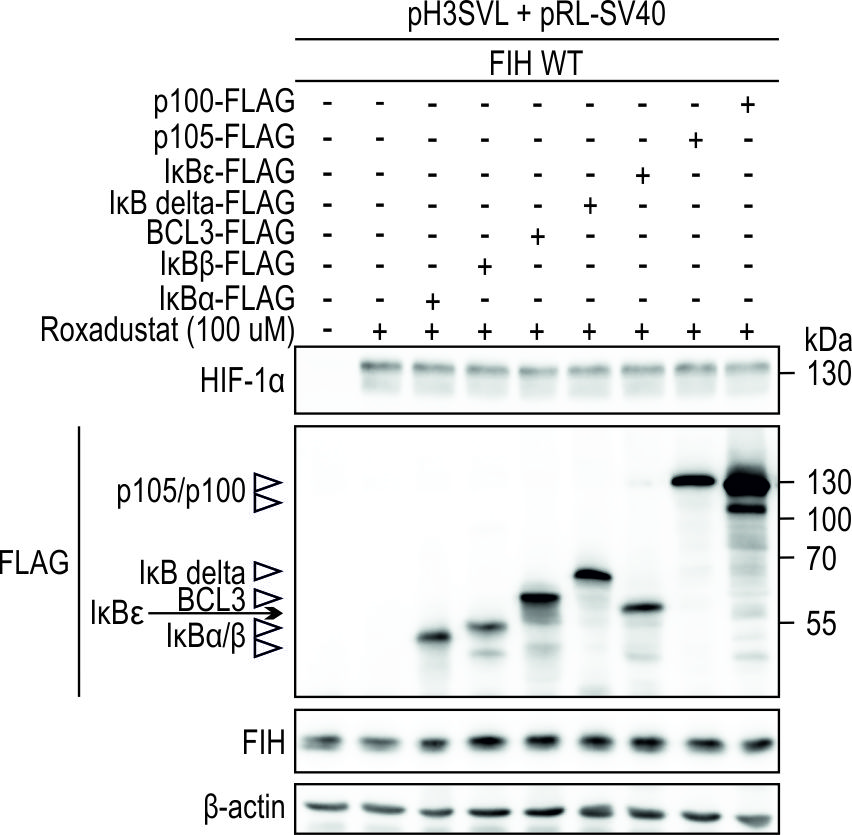


Figure S5. Confirmation of ectopic expression of IκB family members. Immunoblot detection of the ectopic expression of the indicated FLAG-tagged members of the IκB protein family, HIF-1α and FIH, of the experiment depicted in Figure 5A. Cells were harvested 48 h after transfection. Roxadustat treatment was applied for 24 h. β-Actin served as loading control. Data are representative of three independent experiments.

## SUPPLEMENTAL REFERENCES

1. Pickel C, Gunter J, Ruiz-Serrano A, Spielmann P, Fabrizio JA, Wolski W, Peet DJ, Wenger RH, Scholz CC. Oxygen-dependent bond formation with FIH regulates the activity of the client protein OTUB1. *Redox Biol*. 2019;26:101265. doi: 10.1016/j.redox.2019.101265.

2. Kumbhar R, Vidal-Eychenié S, Kontopoulos DG, Larroque M, Larroque C, Basbous J, Kossida S, Ribeyre C, Constantinou A. Recruitment of ubiquitin-activating enzyme UBA1 to DNA by poly(ADP-ribose) promotes ATR signalling. *Life Sci Alliance*. 2018;1:e201800096. doi: 10.26508/lsa.201800096.

3. Lando D, Peet DJ, Gorman JJ, Whelan DA, Whitelaw ML, Bruick RK. FIH-1 is an asparaginyl hydroxylase enzyme that regulates the transcriptional activity of hypoxia-inducible factor. *Genes & Development*. 2002;16:1466-1471. doi: 10.1101/gad.991402.

4. Mader J, Huber J, Bonn F, Dotsch V, Rogov VV, Bremm A. Oxygen-dependent asparagine hydroxylation of the ubiquitin-associated (UBA) domain in Cezanne regulates ubiquitin binding. *J Biol Chem*. 2020;295:2160-2174. doi: 10.1074/jbc.RA119.010315.

5. Stael S, Miller LP, Fernandez-Fernandez AD, Van Breusegem F. Detection of damage-activated metacaspase activity by western blot in plants. *Methods Mol Biol*. 2022;2447:127-137. doi: 10.1007/978-1-0716-2079-3_11.

6. Yu F, Haynes SE, Nesvizhskii AI. IonQuant Enables Accurate and Sensitive Label-Free Quantification With FDR-Controlled Match-Between-Runs. *Mol Cell Proteomics*. 2021;20:100077. doi: 10.1016/j.mcpro.2021.100077.

7. Mirdita M, Schutze K, Moriwaki Y, Heo L, Ovchinnikov S, Steinegger M. ColabFold: making protein folding accessible to all. *Nat Methods*. 2022;19:679-682. doi: 10.1038/s41592-022-01488-1.
